# Extracts and Gleanings

**Published:** 1873-02

**Authors:** 


### EXTRACTS AND GLEANINGS FROM OUR EXCHANGES.

### *MULTUM IN PARVO.*

A Good Derivative Liniment.—During the past two seasons we have used, with much satisfaction, the subjoined liminent, as a derivative and counter-irritant in pneumonia and throat affections—in fact, in all internal congestions and inflammations:

℞.—Coal oil, two ounces; spirits turpentine, one ounce; yolk of one egg; rose or common water, two and a half ounces; acetic acid, half ounce; chloroform, one ounce; camphor, two drachms. M. After rubbing together well the first three articles, transfer to a bottle and add the water and acid, and lastly the chloroform, after dissolving in it the camphor. Shake well occasionally for twenty-four hours. May be used as any other liniment, by smartly rubbing the affected part; but to insure its full derivative effects in pneumonia or other deep-seated inflammations of adults, saturate a woolen cloth with the liniment, apply and cover with a hot mush poultice. G. D. Hodge, M.D.

Holly Springs, Arkansas.

New Theory of Gout.—Dr. A. Meldon says: The predisposing cause of gout is undoubtedly the presence in the blood of uric acid, and of soda in some form; nerve-force, I believe, when in a healthy condition, preserves these two in a fluid state, separately, in a condition in which they may be eliminated by the skin, kindeys or bowels. As soon, however, as this nerve-influence is lessened, these two substances unite in the tissues most removed from the brain and centre of circulation. Irritation and imflammation excite the nervous system to increased energy, and the disease for the time is arrested; often, however, a like cause produces a second exacerbation, a third, or even a fourth, and then Nature gradually alters or removes that which has been deposited, and all traces of the fit have passed away. The time that an attack generally commences is at night, when both nerve-force and the circulation are weakest; the position, moreover, most usually affected—the great toe—favors my theory; and I need scarcely mention the numerous instances recorded where a fit of gout has been brought on by great nervous depression. Columbus never suffered from the disease until disappointment and ingratitude depressed his whole nervous system. Hundreds of similar cases are familiar to all; politicians and speculators are particularly liable to gout.

The action of colchicum furnishes me with one more proof. By an experiment which may be repeated by any one at will, I have satisfied myself that it is a nervous stimulant. Repeatedly have I, while fasting and in perfect health, taken doses of from ten to fifteen minims of tincture of the seeds of colchicum. Its effect was to produce, first nausea, and then increased action of all the organs of the body; the skin became moist, the action of the kidneys and liver increased, and the mental faculties were invigorated. On some occasions the heart's action was much increased, and I have even experienced severe palpitation consequent on its use. All these results can only be produced by a nervous stimulant. This is the only way, too, in which its apparently magical influence on a seizure of the gout can be explained, and it accounts, also, for the injury which its too frequent use may produce.

In conclusion, I have to recommend for trial, in the treatment of chronic gout, some medicines from which I have, in my practice, derived the greatest assistance.

Sulphate of nickel and the triple phosphate of iron, quinine and strychnia will be found of the greatest benefit.

There is but one other point connected with this subject which I desire to mention. It has often been doubted that the skin can eliminate uric acid; but, if a large quantity of perspiration be collected, and, after evaporation, tested, abundant crystals will be obtained. For this experiment it will be necessary, in the first instance, to have a large quantity; I have repeatedly obtained as much as a pint, by aid of the Turkish bath. From a small amount of perspiration I rarely succeed in obtaining positive proofs of its existence.—*Lancet.*

SUBCLAVIAN ANEURISM CURED BY ERGOTINE AND COMPRESSION.—The *Gazette Hebdomadaire,* October 18, mentions a case as having occurred in Berne, of aneurism of the left subclavian, of rapid growth, for which fifteen injections of ergotine were given in the space of six weeks. After the fourth injection there was a notable diminution of the tumor. Digital compression was then practiced for six days, and five months later there were no signs of the disease, objective or subjective.—*Pacific Medical and Surgical Journal.*

Disease of Ear Attributed to Quinia.—In the *Transactions American Otological Society*, recently published, is described the case of a physician who was cinchonized for malarious disease, and who afterwards suffered from violent disease of the ear. His trouble began with laryngeal inflammation and chronic naso-pharyngeal catarrh. When the malarious affection supervened, he took twenty-five grains quinia every other day, until ringing in the ears and dizziness came on, followed by severe otitis. The otitis was subdued by antiphlogistic treatment, and facial neuralgia followed, for which he took fifteen grains quinia, with the effect of producing pain in the ears. Every repetition of the dose was followed by pain. The naso-pharyngeal disease increased, with returning neuralgia and otitis. The last was brought on in twenty-four hours by a dose of quinia. He suffered for several years, spending a portion of the time in Europe. He became deaf, and his ears filled with inspissated wax. At length, after long and varied torment, he was restored to tolerable health by judicious treatment. Dr. St. John Roosa, of New York, who describes the case, attributes much of the ear disease to the quinia.—*Pacific Medical and Surgical Journal.*

Chloral Hydrate in Tetanus Neonatorum.—Dr. W. G. Thomas, of Washington, North Carolina, (*Richmond and Louisville Medical Journal*), gave chloral hydrate in a case of traumatic tetanus, with hypodermic injection of morphia, with successful result, though it was necessary to prolong the treatment for thirty days. During that period twelve hundred grains of chloral was administered. He also succeeded in a case of tetanus neonatorum, by injecting two grains of chloral in the rectum every four hours, "the controlling influence being very manifest after ten days." The treatment was not entirely abandoned till twenty-three days. Some irritation of the rectum was relieved by adding a drop of laudanum occasionally to the enema. Dr. Thomas adds that this was the only case of tetanus neonatorum which he ever cured.

Cathartics in Albuminuria.—Dr. Moore, of Rochester, New York, (*Medical and Surgical Reporter*), read a paper before the Central New York Medical Society, in which the idea was enforced that albuminuria might remain as an organic disease for a considerable length of time; and during that time there was a remedy in sulphate of magnesia. A case was presented, in which the condition of albuminuria was removed by the cathartic treatment. Drs. Benedict and Campbell gave histories of cases which had been managed according to Dr. Moore's plan, which afforded relief. In answer to Dr. Morris, whether the saline treatment had been successful after scarlet fever, Dr. Moore replied affirmatively.

Wurm on the Diphthria Question.—*Blatt. f. Heilwiss.*, 7, 72.—Dr. Wurm makes the following remarks: "It is unfortunately incomprehensible to me, after an experience wanting neither in quality nor quantity, how physicians of celebrity can repudiate the sovereign means of timely, energetic and persistent cauterizations in diphtheria, instead of giving this means the greatest possible popularity." As Dr. Wurm's thesis contains nothing otherwise essential, we will merely give his opinions on this point: He has until now, without exception, always effected a rapid convalescence by means of timely cauterizations with muriatic acid, which were repeated every one to three hours. In the "murderous epidemic," all non-cauterized children died, while three which were "touched" are well to-day. There follows the application, apparently as a result of it, a striking remission and euphoria, with moderation of the pulse, etc. Dr. Wurm complains that he has not time to give more than a glimpse of the clinical course of each case, as he has three hundred bath-guests to attend from 5 A.M. to 10 P.M. He has only found time to affirm the above proposition with great earnestness, because three children who were "touched" are alive and well to-day.—*New York Medical Journal.*

Calabar Beans for Constipation.—Calabar beans have, until lately, been but little used except for contracting the pupil of the eye, and by Dr. Ogle in chorea. Dr. Victor Subbotin, in the *Deutsch. Archiv. Klin. Med.*, proposes them as a remedy for atony of the bowel, observing that it has been found by Bauer, and confirmed by Besold and others, that this drug produces a cramp-like condition in organs that are supplied with involuntary muscular fibres. He uses the following formula: ℞.—Ext. sem. physostig. ven., grs. iv.; glycerin. pur., ℥ ij. Solve. Sig. four drops four times daily. He first administered this solution to a female who was much constipated from atony of the bowel. After the patient had used it twice, a swelling of the abdomen, caused by fæcal matter, disappeared permanently. Aloes and rhubarb had previously given temporary relief. The Doctor has since treated successfully another case of the same nature, and one of chronic bronchial catarrh.—*New York Medical Record.*

Carbolic Acid in Burns.—Dr. J. H. Pooley says: The application of carbolic acid, mixed with some oleaginous material, is of the greatest efficacy in relieving pain; indeed, this application far surpasses any other that I have ever tried for this purpose. One part of pure carbolic acid to seven or eight of olive oil will annul the severe pain of an ordinary burn almost instantly and as if by magic, another practical and useful application of this comparatively new agent.—*New York Medical Journal.*

On Cancer of the Sublingual Glands.—"I am about to direct your attention to a frequent and grave affection: epithelioma, or cancer of the sublingual glands. The subjects of this affection generally come to us with ulcerations which have been cauterized and tormented for several months, and in such a manner as to render surgical intervention difficult. Authors scarcely allude to cancer of the sublingual glands. I have seen a dozen cases of this affection. The affection is generally presented in the form of sharply-cut linear ulceration at the floor of the mouth. The base is indurated; the ulceration extends sometimes towards the lingual mucous membrane, sometimes towards the periosteum. Difficulty in deglutition, salivation, pain radiating to the neighboring parts, even to the ear, are the chief symptoms; this extension of pain is probably due to exposure of the inferior maxillary nerve. All kinds of caustic agents have been applied to these ulcerations. The diagnosis is easy. Aphthous or syphilitic ulcerations do not occupy this position, and have not indurated bases. I have had occasion to study this affection anatomically, and have observed that the seat of the disease is the sublingual glands; the acini are increased to thrice their normal size, the epithelium is altered, all the cells present large nuclei, each containing three bright nucleoli. The prognosis is extremely serious. I have seen recoveries from epithelioma of the tongue and of the inner surfaces of the cheeks, but when the sublingual gland is the seat of the disease, relapse occurs rapidly after operation, even before the cicatrization of the wound. This is one of the most frequently fatal forms of epithelioma. I have performed several operations; have enucleated the tumor and removed it in different ways by means of the écraseur, but all to no purpose; relapses were speedy and death rapid."—*Half-Yearly Abstract of the Medical Sciences.*

Good Effects of Aconite in Acute Pneumonia.—Among other cases of interest which came under the observation of Dr. Murchison, in the male wards of St. Thomas' Hospital, London, was that of a boy aged fifteen, who was admitted with acute pleuro-pneumonia of the right side, and herpes labialis, and considerable increase of temperature. On the administration of eight-minim doses of tincture of aconite, with liquor ammoniæ acetatis every four hours, the temperature at once came down, and the disease did not increase. This quantity was large.—Eds.—*British Med. Jour.*

A Substitute for Quinine.—The last number of the *Gazette Med. de Paris* (September 21, 1872), details a number of cases of intermittent fever treated by the carbazotate of ammonia. The results were as successful and as speedy as with the administration of quinia. The remedy is given in pills in the dose of two ctgr. a day.

Effects of Various Liquors on the Kidneys and Bladder.—Kraus, of Vienna, says, speaking of this subject: "Sparkling wines are very injurious in diseases of these organs, but not on account of their carbonic acid, which assists materially in the elimination of the phosphates. Champagne not only increases the secretions, but, in an extraordinary manner, the phosphates; and the conduct of medical men who advise its use in cases of calculus is very reprehensible. His experience absolutely contradicts the idea of the solvent action of carbonic acid in concretions already formed. Old beer he considers unobjectionable, but new beer is to be avoided, because the fermenting principle enters the mucous membrane and gives origin to a greater or less degree of catarrh. English pale ale is open to the same objection on account of its richness in alcohol and carbonic acid; porter, however, if of good quality and age, he considers to be also unobjectionable.

Digitalis an Anaphrodisiac.—M. Gourvat, in the course of a paper published in the *Gaz. Med. de Paris*, on the action of digitalis, says: "When digitalis, or digitaline, is administered for some time to a man in full possession of sexual powers, these become gradually weakened, the propensities disappear, formation of the liquor seminis diminishes, and may at last cease altogether. The anaphrodisiac properties of the drug are the secret of its geod effect in spermatorrhœa. With women, digitalis or digitaline excites strong, regular and intermittent uterine contractions, and controls metrorrhagia; hence, digitalis is employed in producing abortions. (Tardieu.) It is probable that this drug acts as an anaphrodisiac in women, inducing, by long-continued use, impotence and sterility, appearing also, in their cases, to interfere with the development of the Graafian vesicles; the propagation of species being thus doubly retarded.

Aconite Poisoning.—Dr. Stephen S. Keene, Providence, R. I., (*Boston Medical and Surgical Journal*), publishes a case of poisoning by the *external application* of aconite. For pain in right side of the face, he applied a portion of the following mixture to the affected part by rubbing with the fingers: ℞.—Tinct. rad. aconi, tinct. opii, aa. ℥ ss. M. One-half hour afterwards he was seized with dizziness, nausea, dimness of sight, cephalagia, pain in back, with sensation of coldness running along the spine, etc. One hour after the application, the results from an overdose of aconite internally were exhibited. On examination, no abrasions were discovered on face, but the index finger of right hand had a slight wound. The patient, male, aged forty-five, of good constitution, was treated with ammonia and chloroform, and at the end of forty-eight hours the symptoms subsided.

Conjugal Onanism.—In an important clinical lecture on "Conjugal Onanism and Kindred Sins," by William Goodell, M.D., Clinical Lecturer on the Diseases of Women and Children, University of Pennsylvania, (*Philadelphia Medical Times*), the resulting disorders from imperfect sexual congress, on the part of man and wife, are plainly discussed. He says the husband suffers mentally, because no *man* can behave in so unmanly a way without a keen sense of self-abasement, without being stung by the chastisement of remorse. The wife suffers the most, for she both sins and is sinned against. She sins, because she shirks those responsibilities for which she was created. She is sinned against, because she is defrauded of her rights. The excessive stimulation of the whole reproductive apparatus remains unappeased. Excessive coitus, even when normally performed, and especially indulged in during the fatigue and discomforts of the honey-moon journey, so often the starting-point of uterine disease, with disorders resulting therefrom, were alluded to. Such papers are especially needed at the present time, as some members of prominent societies, like the "London Dialectical Society," have been earnestly advocating plausible arguments against "Over-Population and Large Families."

Carbolic Acid and Yellow Fever.—The New Orleans Health Officers have great faith in carbolic acid as a preventive of yellow fever. It appears from their published report that ten thousand gallons were distributed through a single district in three days, on the occasion of an outbreak of the disease. "The entire vicinity was soaked with it," says the report. The atmosphere was so highly charged that the fumes penetrated all the houses alike. Many persons "fancied" they had headache from the fumes, but the generous distributors of carbolic acid corrected the erroneous impression.—*Pacific Medical and Surgical Journal.*

Maltine.—This substance, called *diastase*, first reported by Payen and Persoz in 1833, is recommended in dyspepsia, either with or without pepsine, by Professor D. Renzi. The subjoined formula is laid down in *La Nuova Liguria Medicale:* ℞.—Maltine, centigr. xv. F. pil. No. 3. One pill before each meal. ℞. Maltine, centigr. v. to x.; pepsine, centigr. c. to cl. F. doses No. 2.

Stramonium an Antidote to Opium Poison.—In two cases, Dr. D. B. Putnam, of Boston, Massachusetts, (*Boston Medical and Surgical Journal*), has used stramonium as an antidote to poisoning by opium, and from the promptness and certainty of effect manifested in these cases, he would suggest to others a trial of its virtues in cases of this character.

Inhalations of Bromine in Diphtheria and Croup.—The fact, says Dr. Schutz, that diphtheritic membranes are more readily soluble in a solution of bromide of potassium than in lime water, or other substances usually employed in the treatment of diphtheria, induced the writer, some years ago, to adopt inhalations of bromine in the treatment of this disease. His success therewith has been so good that he again, in some recent numbers of the *Wiener Med. Wochenschrift*, urgently commends it to the notice of the profession. He advises the use of a solution of pure bromine and bromide of potassium, each three-tenths of a gramme, to water one hundred and fifty grammes. A sponge is soaked in this solution, placed in a tunnel of stiff paper, and held over the nose and mouth for inhalation, just as is done with ether or chloroform, the inhalation being continued for five or ten minutes, and repeated every half-hour or hour. The odor of bromine, as diluted, is very well borne even by infants. The preparation being highly volatile and decomposed by light, must be guarded accordingly.—*Allg. Med. Central-Zeitung.*

Hesitation in Curing Eczema.—Mr. Milton, (*Medical Press and Circular*), for years has done his best, in every instance, to check the discharge of eczema as quickly as possible. During that period above five thousand cases have passed under his notice. He concludes that the fear of curing eczema, of however long standing it may be, and however delicate the health of the patient, is not warranted by either proof or analogy; that no known agent possesses the power of repelling eczema; that we can cure it only by means which improve the health at the same time; and that it is as justifiable to arrest its discharge as that of diarrhœa or cholera. All that has been said of eczema may be said of ulcer; there is no danger of healing it up, no bad symptoms ever arose from doing so.

Large Pleuritic Effusion Treated by Thirst.—A case in which this mode of treatment was pursued by Dr. Moxon, in Guy's Hospital, is reported in the *Medical Times and Gazette* of January 6th. At the commencement of the treatment, the pleural cavity was filled with liquid. The drinks of the patient were limited to ten fluid ounces per diem, and five grains each of iodide of potassium and muriate of ammonia were given three times daily. By the middle of the third week of treatment, dullness on the side had disappeared.

Syphilis.—℞. Fld. ext. stillingia, ℥ ij.; fld. ext. phytolacca, dec. ℥ j.; fld. ext. corydalis, form. ℥ jss.; potass. iodide, ℥ j.; syr. simplex, ℥ iijss. M. S. Dose, one teaspoonful three times per day, largely diluted with water. The dose may be gradually increased to two teaspoonsful.

A Simple Method of Arresting Epistaxis.—Dr. Roland G. Curtin says (*Philadelphia Medical Times*) Dr. Albert H. Smith, in order to soften the nasal mucus of children in the nostrils, recommends the introduction of lard upon a small roll of fine linen wrapped like an ordinary lamp-lighter. It occurred to me, in a case of epistaxis, that a similar roll of paper, moistened with water and coated with the dry tannic acid, inserted into the nose, might be of service. I tried it, with immediate success. I have since found that old linen answers the purpose better than paper applied as above, as it makes a better carrier, being softer, more flexible, and less liable to break down through excess of moisture. I have also found that the powder adheres better if soft lard be used instead of water. I have tried this repeatedly with uniform success, and believe, if it were resorted to, that the disagreeable operation of plugging would seldom be found necessary.—*New Remedies.*

Treatment of Croup.—Dr. Daquillon recommends (*Gazette Hebdomodaire*) the following treatment when symptoms of suffocation from croup are imminent: A sponge, after being dipped in pure liquor ammonia, is to be squeezed until no more liquid drops from it, and is then to be passed, by means of a whale-bone probang, down the throat. The child should be compelled to inhale the vapor of ammonia as long as it possibly can. The immediate result of this treatment is an abundant secretion from the respiratory mucous membrane and the expectoration of false membrane; the dyspnœa diminishes, the cough is less hollow, and the voice is less muffled. Often, in cases where death seemed imminent, the lives of children have been saved.—*Medical Times.*

A Singular Way of Opening the Bowels.—In old times they had a pill called "*Peristaltic Persuader*," which was much relied on for the relief of habitual constipation. A writer in the Philadelphia *Medical Reporter* proposes to accomplish the purpose by what might be called tapping at the door. He says: At a regular hour every day, all things being in readiness, tap gently and repeatedly on the anus with a bit of wood or any substance hard enough to produce irritation, and the sphincter will almost certainly relax after a while.—*Pacific Medical Journal.*

Chalk Mixture.—George W. Kennedy (*American Journal of Pharmacy*) recommends the following formula: ℞.—Cretæ præpt., glycerinæ (Bowers'), aa. ℥ ss.; pulv. acaciæ, ʒ ij.; olei cinnamoni, gtt. viij.; aquæ destill., ℥ viij. Mix thoroughly. The above mixture I have kept a whole summer and up to the present time; I made it about ten months ago, and upon opening it I found it in perfect condition, not even the slightest acidification having taken place.

Varicocele.—The object of treatment of varicocele is the obliteration of the mass of veins of which it consists; i. e., when the case is so bad as to require operative interference at all. Mr. Wood, of King's College Hospital, recommends continuous elastic wire pressure for this purpose, the elasticity being obtained by means of the instrument used. The instrument consists of two limbs united by a strong spring at one extremity. One limb ends in a thin round steel shaft, joining the limb at right angles, and terminating in a transversely oval and obliquely placed eye. The loop of wire having been made to surround the veins is passed through this eye, and the free ends are twisted round the end of the other shaft, which is first made to approximate its fellow. The spring will now cause constant tension upon the wire surrounding the veins.—*British Medical Journal.*

[The ordinary plan of obliterating these veins is by passing two or three small hair-lip pins underneath them, and winding a figure of eight ligature round the free ends of the pins.]—*Braithwaite.*

Consumption.—Dr. McCormac, of London, in his pamphlet on consumption, remarks as follows: The habitual respiration of air not pre-respired is essential, absolutely, to the effective prevention of consumption, scrofula, and other forms of tubercular disease. Air pre-breathed will not sustain combustion, will not sustain life. About forty grains of effete carbon are excreted every fifteen minutes, in the form of carbonic acid gas, from the lungs, provided always that air *not* pre-breathed shall be alone respired. If not, the effete carbon, being insufficiently oxidized, is retained *pro rata* as tubercle within the living organism, and leads sooner or later to its destruction.—*Canada Lancet.*

Surgical Treatment of Anasarca.—In a severe case of anasarca, Dr. Wolff (*Berlin. Klin. Wochenschr.*) introduced into the skin a number of fine cannulæ, such as are used for subcutaneous injection, and left them there. Through twenty-five cannulæ thus used, twenty quarts of fluid were discharged in three days. The fluid was carried away in elastic tubes into a vessel near the bed. No inflammation of the punctures, nor any other unpleasant results, followed the operation.—*Clinic.*

Catarrh.—Take of carbolic acid (crystals) four scruples; caustic liquor ammonia one fluid drachm and a half; rectified alcohol three fluid drachms; distilled water two fluid drachms; mix. Fill a wide mouthed vial of sufficient capacity with small pieces of sponge moistened with the mixture, and then fill the vial with the balance of it. This, in ordinary catarrh, has been found a very valuable and efficient remedy. All that is required is to uncork it several times a day and smell or inhale the vapor for a few minutes each time.

Stuttering Relieved by an Artificial Palate.—The following case of stuttering cured by an artificial palate may not be without interest to the readers of the *Journal:* William D., a clerk, aged nineteen, was admitted an out-patient at St. Clement's Hospital, Leamington, under the care of Dr. Horace Leete. He had stammered from childhood, but lately so much that he could not retain any situation. After a thorough examination, Dr. Leete came to the conclusion that the primary cause of the stuttering was the abnormally high palate. He found that when the patient attempted to articulate those words necessitating the use of the tongue against the palate he failed, and so lost confidence in himself in speaking other words. Unless the teeth were quite closed, the palate could not be reached by the tongue. Feeling sure that this was the cause, Dr. Leete referred the case to the dental department of the hospital, and a suction plate of vulcanite was fitted three-eighths of an inch in thickness, scooped away underneath to form a large suction cavity and also to reduce the weight. At the end of three days his speech was much better, and it continued to improve up to the time of his discharge, when he spoke without hesitation.—*W. C. Williams, L.D.S., in British Journal of Dental Science.—Dental Cosmos.*

Management of Obstinate Pruritus.—Mrs. J. G. Brown, Resident Physician Ill. Woman's Hospital, (*Med. Exam.*, Aug. 15, 1872), speaks highly of the sulpho-carbolate of zinc in cases of obstinate pruritus of the vulva. In one particular case, the vulva, after being thoroughly bathed with tepid water, was washed twice daily with a solution of the sulpho-carbolate of zinc containing half a drachm to the ounce of distilled water, the parts being allowed to dry without wiping. She was much improved at the end of a week. During the second week the application was made once daily, on retiring at night. At the end of the third week the pruritus was wholly relieved.

Amaurosis.—In a case of amauroses the ophthalmoscope showed dullness of the media, anæmia of the disc, retina pale and anæmic. Total amaurosis had existed for nine months. The twenty-fourth of a grain of strychnia was injected, and the next day the patient could count fingers and distinguish colors. A continuance of the treatment led to almost complete recovery of sight.—*Braithwaite's Retrospect.*

Cystitis.—Take of tincture of iodine from one to three parts; iodide of potassium one part; water five hundred parts; mix. Inject daily into the bladder three and a half fluid ounces of this mixture. When the cystitis is quite painful, extract of belladonna, one part, should be added to the above.—*Mallez.*

IODIDE OF CALCIUM.—Dr. E. M. Corson (*Trans. Pa. State Med. Soc.*) "claims for it superiority over the iodide of potassium on several accounts. 1. It contains more iodine in a given weight than is contained in a like weight of iodide of potassium. 2. It is less liable to derange digestion than salts having a potash base. 3. The irritability of stomach, nausea, and vomiting or purging which sometimes occur from the use of iodide of potassium, being probably due, he thinks, to the carbonate of potassa with which the iodide is combined (and not to the iodine), and no such objectionable combination existing in the iodide of calcium, it is therefore to be preferred. 4. As lime is useful in tuberculous diseases, and could be advantageously given with cod-liver oil, the iodide of calcium is to be preferred, and more especially as he has found that the syrup of iodide of calcium forms a perfect emulsion with cod-liver oil, which does not separate on standing; and the disagreeable taste of the oil is by the combination in a great measure destroyed. The syrup of iodide of potassium forms no such emulsion with the oil."—*Medical Cosmos.*

USE OF MILK IN CANCER OF STOMACH.—The invaluable benefit of milk diet in cases of cancer of the stomach has been forcibly brought out in an instance recorded by the *France Médicale* for August 24. The patient, under the care of Dr. Siredey, at the Hôpital la Riboisiére, had not been able for two months to take any kind of food without immediately throwing it up. Milk, in small quantities at first, was then ordered as diet. It was not brought up, and consequently during thirty-six days it was used in any quantities, and without inducing sickness. At the end of this time other sorts of food were given and properly retained.—*Lancet,* August 31, 1872.

GONORRHŒA.—℞. Copaiba, spts. ether nit., aa. ℥j.; ol. cubeb, ℥ss.; tinct. opii camph., ʒvj.; tinct. aconite, gtts. xx. M. S. Dose, one teaspoonful three times per day. I have had a very extensive practice in the treatment of gonorrhœa, with uniform success, and depend upon the above treatment, adding to it veratrum, gelseminum, aconite or belladonna, as the febrile or inflammatory symptoms seem to demand. I never use injections under any circumstances.—*Eclectic Medical Journal.*

DELIRIUM TREMENS.—An excellent prescription for delirium tremens is twenty grains of bromide of potassium, with two drachms of the compound elixir of symbul, in an ounce of water, every two or three hours. The bowels should be opened at the same time with comp. cath. pills and good rich soup, seasoned with pepper, given freely. Chloral may be given at night to induce sleep.—*Physician and Surgeon.*

Morphia Antidotal to Atropia.—M. Abeille communicated a case to the Academy of Medicine, in which symptoms of poisoning induced by the hypodermic injection of nine milligrammes of atropia were treated and cured by the injection of thirty-seven milligrammes of the hydrochlorate of morphia, without any other means (not even sinapisms) being had recourse to. It was a case of sciatica, which during four months had resisted all means of treatment, including morphia injections. Having in similar cases obtained successful results from atropia, M. Abeille resolved on its use, notwithstanding its well-known danger, being satisfied that he could meet this by means of morphia. This conviction was the result of its use in a case of very dangerous poisoning related to the Academy in 1868. In the present case the cure was complete, for it has continued during two years, and the symptoms of poisoning were dissipated within three-quarters of an hour by one injection of morphia.—*Bullet. de l' Acad.*

The Bowel Lesion of Typhoid Fever.—The generally entertained opinion that the bowel lesion is the result of Nature's efforts to eliminate, is entirely erroneous. Were this true bowel lesion, it would relieve rather than aggravate the constitutional symptoms. The inflammation of the agminated and solitary glands bears exactly the same relation to the fever that the sore throat of scarlet fever does to that disease, that is, it is the direct effect of it. No doubt the sloughs and discharges from the ulcerated glands carry the poison of typhoid fever and are capable of conveying the disease from one person to another, just as the discharges from the mouth and nostrils in scarlatina are capable of transmitting their peculiar poison.—*Braithwaite.*

Varicose Veins.—In a paper read before the Central Kentucky Medical Association, by A. D. Price, M.D., (*Richmond and Louisville Medical Journal*, August, 1872), two obstinate cases of varicose veins successfully treated with injections of persulphate of iron were detailed. The author remarked that this method has at least three advantages over all others; that it is easily performed, and need give but little pain; and that the only instrument necessary is an ordinary hypodermic syringe.

Pruritus Vulvæ.—Those who have had any experience in this oft-times most troublesome affection will be pleased to learn that Mr. McGraith announces, in the *Canada Lancet*, that he has found, from the following solution: ℞.—Biborate of soda, gr. ij.; hydrochlorate of morphia, gr. xx.; hydrocyanic acid, gr. j.; glycerin, f℥j.; distilled rose water, f℥vij.; applied with a sponge, morning and evening, after ablution, speedy and most satisfactory results.

Palliative Treatment of Ectropion.—In a clinical lecture published by the *Presse Médicale Belge*, Dr. Thiry lays great stress on the palliative treatment of ectropion, and the very successful results which he had obtained by means of the subnitrate of bismuth. In combating the frequent inflammations of the palpebral conjunctiva so often met with in the course of ectropion, as also the spasms which depend upon these inflammations, and which constantly tend to increase the eversion of the eyelid, Dr. Thiry has employed with great benefit the following preparation: Subnitrate of bismuth, one drachm; starch, two drachms; glycerin, three drachms; mix. Fifteen to thirty grains of acetate of lead may be added when there is need to exert an astringent action on the tissues.

Diagnostic Sign of Cancer of the Neck of the Womb.—Besides the difference as to consistence between cancer and simple induration of the neck of the uterus, Professor Spiegelberg, of Breslau, gives the following sign as distinctive: "The mucus in cancer is firmly adherent to the subjacent tumor, and immovable, while the contrary is the case in hyperplastic thickening and induration. In the latter condition, the mucus, under the influence of compressed sponge, becomes looser, softer and thicker. In cancer it invariably remains hard and rigid, and cannot be torn."—*La France Medicale.—Chicago Medical Examiner.*

Heart Affections.—Digitalis is not merely a temporary remedy in cases of insufficient power of the muscular walls of the heart. It may be given continuously and uninterruptedly for years with excellent effect. With this, however, we should not forget the great necessity and advantage of rest, and improvement of the nutrition of the heart itself by iron, cod-liver oil, arsenic, and other remedies which increase blood formation.—*Braithwaite's.*

Hiccup.—Dr. Wolf has frequently succeeded in arresting the hiccup which supervenes in the course of disease or under other circumstances, by causing the patient to take a full inspiration and to strain with the abdominal muscles as if he were at stool, and remain in this state, without breathing, as long as possible, and when he does breathe, to take a very rapid inspiration. The process must be repeated several times before it is successful.

Cure for Corns.—A correspondent of the *Country Gentleman* states that he has found the following a simple and effectual remedy for corns: "Bathe the feet in tepid water, to soften the corns; pare these off very closely with a sharp knife; then rub on well green peach-tree leaves, when, after continuing the rubbing once or twice a day, the corns will disappear.

Treatment of Asthma.—George Goskoin, Surgeon to British Hospital for Diseases of the Skin, has had success in treating this disease by rubbing briskly into the chest, for the space of an hour, a chloroform liniment. The counter-irritation produced by the liniment was of benefit, but this benefit was increased by the jolting resulting from the rubbing. Anything that leads to the displacement of the air stagnated in the vesicles has proved able to relieve in many instances. It is advised that the friction be made with as much roughness as the case admits. Slight blows with the palm of the hand or the end of a towel on the ribs are quite allowable; and the friction should be extended to the front of the neck at the lower part, where the vagi enter the chest. The composition of the liniment need not trouble us, provided it be warm and work well.—*British Medical Journal.*

Carbolic Acid in Small Pox.—Dr. A. Loffler states in a Vienna medical journal that he has treated more than forty cases of small pox by the external copious application, by means of cotton wool, of a solution of one part of carbolic acid in twelve of oil. The result in all cases was, that the cutaneous swelling soon diminished, and that, when the application was made early, the course of the disease, in relation to the number of pustules, was milder. He believes, also, that by this treatment the danger of infection was diminished.—*Archives.*

Hæmorrhoidal Tumors of the Female Urethra.—The *Journal de Med. et de Chir. Pratique* contains an article by M. Richet, on this affection, in which this gentleman calls attention to the fact that many of the painful tumors found in this locality are hæmorrhoidal, and not, as is sometimes supposed, polypoid growths from the mucous membrane. The galvanic cautery and forcible dilatation are spoken of as the most successful modes of treatment. *New York Medical Record.*

A Good Local Application in Erysipelas.—Alum reduced to impalpable powder, thirty parts; white precipitate, one part. Rub up well together, and place the powder in a bottle, and then add from ninety to one hundred parts of glycerin. Shake the bottle until the mixture assumes a creamy consistence, and repeat the shaking whenever the application is about to be employed.

Chronic Conjunctivitis.—The best strength for a solution of nitrate of silver is ten grains to the ounce. Mr. Liebreich, at St. Thomas' Hospital, relies almost exclusively on the daily application of this solution in the treatment of chronic inflammatory affections of the lids, and the superficial structures of the globe.—*Braithwaite's Retrospect.*

RINGWORM IN SCHOOLS.—Dr. Tilbury Fox reports that in an epidemic of ringworm in a large school, he found the dust of the room to contain abundance of spores of tricophyton, the fungus of the disease. If, therefore, the air of such a room be not thoroughly disinfected by burning sulphur in it, the disease is liable to break out again. Recent cases may be at once checked, and often cured, by simple blistering. If the disease has reached the bottom of the hair follicles, epilation should be practiced. A good plan is to keep the head soaked in a solution of sulphurous acid; of course, a proper cap of silk should be worn. The head should be washed each day. The question frequently has to be decided whether a child is well, and fit to return to school; the best guide is the presence or absence of short broken-off hairs; if these exist, the disease is not cured. The hair ought not to be dull and dry, and the scalp should be free from scurfiness before the child is allowed to return.—*Braithwaite.*

HYDROCELE.—In a clinic lecture Professor Agnew remarked: The next case is a little boy six months old, who has a hydrocele upon the left side. It is now increasing in size and must be arrested. I shall therefore to-day simply puncture the sac, draw off the liquid, and then apply a liniment of muriate of ammonia. If this is not successful, I shall then pass through the sac a simple strand of silk, allowing it to remain *in situ* until it has produced a sufficient amount of inflammation, when it will be withdrawn. In young children this method of cure is usually all-sufficient.

TREATMENT OF DIABETES INSIPIDUS.—M. Gueneau de Musy, in a clinical lecture at the Hôtel-Dieu, recommends the administration of full doses of belladonna and sulphurous baths in the treatment of diabetes insipidus. He has twice found belladonna to accidentally produce anuria. Its use in incontinence of urine is well established. Systematically employed in diabetes insipidus, it has diminished the quantity of urine passed from ten pints to two pints *per diem.* The sulphurous baths bring the skin to the relief of the kidney.—*British Medical Journal.*

PHYTOLACTIA IM MAMMARY INFLAMMATION.—A recent case suggests that we name this special influence of phytolacca again. The inflammation was acute, of twenty-four hours' duration—small doses of aconite and phytolacca alternated; with a local application of one part tincture of phytolacca to lard three parts, and support to the breast, relieved the patient in forty-eight hours.—*Eclectic Medical Journal.*

GOOD FOR CHRONIC RHEUMATISM.—℞. Syr. sarsaparilla, ℥ iii.; tinct. colchici, ℥ i.; hyd. potassa, ʒ iii. M. Thirty drops to be taken three times a day.—*M. ℞.*

Sick Headache.—The only remedies which are of any avail are those which act on the nervous system, such as hot tea and coffee; or, after the stomach is quieter, and the more urgent symptoms have passed off, a little wine or ammonia. If the headache take more the form of hemicrania, then remedies are occasionally useful, as the local application of the bisulphide of carbon, or galvanism, and internally the bromide of potassium. This is the only drug which I have really seen to be serviceable. Whilst the nausea exists and the worst symptoms prevail, even this remedy is of no avail.— *Wilks, British Medical Journal.*

Cancer.—Edwin Payne, M.D., in a letter to the *London Lancet*, says that while search is being made for a cure for cancer, it will not be uninteresting to many of your readers to hear of a palliative. I have used as a lotion with great palliative success, in a case of cancer of the breast, and in another case of cancer of the rectum, an American drug, "Hydrastis Canadensis;" it was preferred by both patients to any other form of lotion, for its painlessness and for its power of keeping the surface in a more healthy condition and free from offensive odor. The strength in which it was used was one drachm of tincture to eight ounces of water.

Veratrum Viride an Antidote to Opium.—Dr. E. H. Sholl, of Alabama, communicates a case of poisoning by morphia, which was cured by veratrum. The patient, a negro boy, aged fifteen years, had typhoid fever, and took an overdose of morphia, which had been prescribed for hiccough. It was followed by stertorous breathing, contracted pupils, and so forth. His mouth was pried open, and gtt. xviij. Norwood's tincture poured in with two ounces of brandy. In one hour, every symptom of poison had vanished.—*Medical and Surgical Reporter.*

Chronic Orchitis Cured with Phytolacca.—John R—— had gonorrhœa some eight months since, followed by orchitis. The testicle continues enlarged from chronic inflammation. Prescribed tinct. phytolacca, ℥ ss.; water, ℥ iv.; a teaspoonful four times a day. The same as a lotion one part to three of water; a suspensory bandage. The recovery was complete in some three weeks.—*Eclectic Medical Journal.*

E. Scheffer, in the *American Journal of Pharmacy* for August, publishes some interesting observations on pepsin. These are some of his conclusions: 1. Pepsin and alcohol are incompatible. 2. Pepsin loses its digestive power in alkaline solutions. 3. Pepsin is precipitated in the presence of bismuth, even when a glycerole of bismuth is first formed. An elixir, therefore, containing pepsin and bismuth cannot be made.

Pepsin Wine in Diarrhœa of Young Children.—Dr. A. Davidson extols (*Practitioner*) the efficacy of pepsin wine in the diarrhœa often observed in children from one to two years old, resulting from feebleness of the digestive powers, in which a large proportion of the food is not assimilated, but passes off in an undigested state. Aromatic and astringent mixtures are useless in these cases, but Dr. D. says that pepsin wine given in doses of a teaspoonful three or four times a day with the food, soon effects a cure. This statement, though it has no claim to novelty, is interesting as confirmatory of the observations of Dr. J. C. Reeve, of Dayton, Ohio, published in the number of this journal for July, 1865, pp. 55-58.—*American Journal of the Medical Sciences.*

Hare-Lip.—The recommendation has recently been revived, that, after the pins or sutures have been removed from a hare-lip which has been operated upon, collodion should be used to draw and keep the parts closely in apposition. Gauze, or a thin layer of cotton wadding, is saturated with collodion, and while the cheeks are pressed together, this is spread over the lip like a mustache. The contractile power of the collodion is sufficient to hold the newly healed parts together with a considerable degree of firmness; and the advantage is thus obtained of being able to remove the sutures early, by the third or fourth day.

Control of Hemorrhage by Ipecac.—Dr. Martin reports to the *New York Medical Journal* a case of capillary hemorrhage, controlled by small doses of ipecac, given until vomiting occurred. The tonsil had been injured, and ordinary styptics not proving efficacious, recourse was had to ipecac. As soon as vomiting occurred, after two-grain doses of the medicine, given every two hours, the hemorrhage was effectually checked.—*Boston Medical and Surgical Journal.*

Catechu and Opium as an Astringent in Gleet.—Dr. R. Locke Johnson extols for the cure of gleet an astringent composed of tinct. opii, ʒ ij.; tinct. catechu, ʒ ss.; mist. acaciæ, ℥ ij. M. To be used twice daily. He relates one case in which the discharge ceased after the second application, and did not return.—*Medical Press and Circular.*

Case of Amaurosis Cured by Electricity.—Dr. Ramorino (*La Nuova Lig. Med., No.* 26) says that he has cured, in his eye dispensary in Genoa, with three applications of the electric current, a youth of twenty-five, a mason, affected with anæsthesia of the retina of the right eye, after a blow from the point of an umbrella. *Doctor.*

Recurring Tonsillitis.—Dr. Lewin, of Berlin, recommends the application of crystallized chromic acid to those cases of tonsillitis in which a chronic congestion exists, constantly liable on the least provocation to blaze up into an acute attack. The acid causes ulceration of the tissue to which it is applied, and induration of the surrounding parts to such an extent as to compress the enlarged capillaries beyond the possibility of their subsequently taking on inflammatory hyperæmic action. In acute painful conditions of these organs, he injects a morphine solution into their substance, and by so doing greatly lessens the pain and difficulty of deglutition.

Surgical Affections of Adolescents.—M. Gasselin, of Paris, offers the following formula for guidance in the selection of means of treatment in this class of persons: "The special, spontaneous surgical affections of young persons have a tendency to persist, increase or relapse, as long as the period of adolescence lasts; but these tendencies are lost as soon as adult age is reached." Thus, he says, ingrowing toe-nail will constantly recur up to the age of twenty-three or twenty-four, but very rarely will do so after the twenty-fifth year. Ostitis, exostoses and fibrous naso-pharyngeal polypi, are cited as other examples of the same rule.

Color of the Vagina in Pregnancy.—Dr. Albert states that of all the signs of pregnancy hitherto known, that derivable from the observation of the dark red color of the vagina is the best, seen as it is by the aid of a speculum at so early a period of pregnancy, and proceeding progressively with the development of the uterus. He has tested its utility in about thirty cases, and has besides examined, with the same success, a great number of animals at various periods of gestation.

Gelseminum in Irritable Bladder.—Dr. W. S. Hill, of Augusta, Maine, (*American Medical Journal*), reports five cases of irritable bladder cured by the fluid extract of gelseminum, of which ten to fifteen drops were given three or four times per day. In some cases it was combined with bromide of potassium. The difficulty and pain in micturition were quite severe in two or three of the patients.—*Pacific Medical and Surgical Journal.*

An Excellent Pill for Dyspepsia and Derangements of the Liver.—Hydrargyri chloridi mitis, pulvis ipecacuanhæ, svapniæ, aa. gr. 1-10; quiniæ sulphatis, gr. 1-5. One, two or three of these can be taken three times a day.

The effect of the constant galvanic current over neuralgic pain is sometimes truly marvelous, whereas the interrupted current is of little or no service.

